# 765 Charting a Path to Enhanced Burn Care: Standardized Note Templates and Resident Perspectives

**DOI:** 10.1093/jbcr/irae036.307

**Published:** 2024-04-17

**Authors:** Nicolas Malkoff, Brigette Cannata, Dania B Johnson, Deborah Choe, Maxwell B Johnson, Justin Gillenwater

**Affiliations:** Keck School of Medicine of USC, Carlsbad, CA; Keck School of Medicine of USC, Staten Island, NY; Keck School of Medicine of USC, Los Angeles, CA; University of Southern California, Los Angeles, CA; Keck Medicine of USC, Los Angeles, CA; Keck School of Medicine of USC, Carlsbad, CA; Keck School of Medicine of USC, Staten Island, NY; Keck School of Medicine of USC, Los Angeles, CA; University of Southern California, Los Angeles, CA; Keck Medicine of USC, Los Angeles, CA; Keck School of Medicine of USC, Carlsbad, CA; Keck School of Medicine of USC, Staten Island, NY; Keck School of Medicine of USC, Los Angeles, CA; University of Southern California, Los Angeles, CA; Keck Medicine of USC, Los Angeles, CA; Keck School of Medicine of USC, Carlsbad, CA; Keck School of Medicine of USC, Staten Island, NY; Keck School of Medicine of USC, Los Angeles, CA; University of Southern California, Los Angeles, CA; Keck Medicine of USC, Los Angeles, CA; Keck School of Medicine of USC, Carlsbad, CA; Keck School of Medicine of USC, Staten Island, NY; Keck School of Medicine of USC, Los Angeles, CA; University of Southern California, Los Angeles, CA; Keck Medicine of USC, Los Angeles, CA; Keck School of Medicine of USC, Carlsbad, CA; Keck School of Medicine of USC, Staten Island, NY; Keck School of Medicine of USC, Los Angeles, CA; University of Southern California, Los Angeles, CA; Keck Medicine of USC, Los Angeles, CA

## Abstract

**Introduction:**

Common Data Elements (CDE) are standardized questions with a set of limited responses that are used systematically across multiple sites. They have been proposed as a means of standardizing clinical documentation and improving interinstitutional data sharing. Our previous work identified 33 CDEs relevant to burn care based on published recommendations and found that they were documented inconsistently in our burn center. Our aim was to assess current charting practices and attitudes toward CDE-focused note templates at our institution as part of a larger effort to standardize and improve clinical documentation quality.

**Methods:**

Four note templates incorporating 33 CDEs were prepared for inpatient history and physical notes (H&P), floor daily progress notes (PN), intensive care unit progress notes (ICU PN), and discharge summary notes (DC). Prior to their implementation, residents who previously rotated through the burn unit from 1/1/2023 to 9/4/2023 were administered a pre-template survey to assess their experiences and attitudes relating to charting with and without templates. Results were summarized using descriptive statistics.

**Results:**

The pre-template survey was completed by 14 residents. The majority reported spending 15-30 minutes on average documenting H&Ps (57%), ICU PNs (57%), and DC summaries (64%), while 86% spent less than 15 minutes documenting floor PNs. When asked to rate the burden of charting, 50% reported a 7 or higher on a 10-point scale. Only 7% were 90% confident or higher that they commonly document all 33 CDEs. Most (57%) claimed to record fewer than 20 of the CDEs on average. Assessing resident attitudes towards note templates, it was found 86% agreed or strongly agreed that they would reduce the time spent and the burden of charting. When asked if templates would improve their wellness, 57% agreed or strongly agreed. While 72% of residents agreed or strongly agreed that templates would help them more thoroughly evaluate patients, only 43% thought note templates would provide educational value.

**Conclusions:**

Current charting practices in our burn unit are time-consuming and burdensome for rotating residents, which may contribute to suboptimal acquisition and reporting of critical data elements. Most residents expressed positive attitudes towards the implementation of standardized note templates. Future directions for this quality improvement initiative include implementing note templates to study the effects this has on the inclusion of CDEs in patient notes and residents' charting experiences.

**Applicability of Research to Practice:**

Readers may consider the methodology and results of this study when evaluating charting practices at their own institutions.

